# The interaction of YBX1 with G3BP1 promotes renal cell carcinoma cell metastasis via YBX1/G3BP1-SPP1- NF-κB signaling axis

**DOI:** 10.1186/s13046-019-1347-0

**Published:** 2019-09-03

**Authors:** Yong Wang, Jing Su, Yiting Wang, Donghe Fu, Justin E. Ideozu, Hua Geng, Qiqi Cui, Chao Wang, Ruibing Chen, Yixi Yu, Yuanjie Niu, Dan Yue

**Affiliations:** 1 The Second Hospital of Tianjin Medical University, Tianjin Institute of Urology and School of Medical Laboratory, Tianjin Medical University, Tianjin, 300070 China; 2 Department of Laboratory Medicine, Children’s Hospital of Hebei Province, Shijiazhuang, 050031 China; 3 Department of Clinical Laboratory, Tianjin Medical University General Hospital, Tianjin Medical University, Tianjin, 300052 China; 4 Ann & Robert H. Lurie Children’s Hospital of Chicago, Chicago, IL 60611 USA; 5 Human Molecular Genetics Program, Stanley Manne Children’s Research Institute, Chicago, IL 60614 USA; 6 Northwestern University Feinberg School of Medicine, Chicago, IL 60611 USA; 7 Center for Intestinal and Liver Inflammation Research, Stanley Manne Children’s Research Institute, Ann & Robert H. Lurie Children’s Hospital of Chicago, Chicago, IL 60611 USA; 8 Department of Pediatrics, Feinberg School of Medicine at Northwestern University Chicago, Chicago, IL 60611 USA; 9 Department of Genetics, School of Basic Medical Sciences, School of Medical Laboratory, Tianjin Medical University, Tianjin, 300070 China; 10 Department of Microbiology, School of Medical Laboratory, Tianjin Medical University, Tianjin, 300070 China

**Keywords:** Cancer, G3BP1, Renal cell carcinoma, SPP1, YBX1

## Abstract

**Background:**

Renal cell carcinoma (RCC) is a deadly urological tumor that remains largely incurable. Our limited understanding of key molecular mechanisms underlying RCC invasion and metastasis has hampered efforts to identify molecular drivers with therapeutic potential. With evidence from our previous study revealing that nuclear overexpression of YBX1 is associated with RCC T stage and metastasis, we investigated the effects of YBX1 in RCC migration, invasion, and adhesion, and then characterized its interaction with RCC-associated proteins G3BP1 and SPP1.

**Methods:**

Renal cancer cell lines, human embryonic kidney cells, and clinical samples were analyzed to investigate the functional role of YBX1 in RCC metastasis. YBX1 knockdown cells were established via lentiviral infection and subjected to adhesion, transwell migration, and invasion assay. Microarray, immunoprecipitation, dual-luciferase reporter assay, and classical biochemical assays were applied to characterize the mechanism of YBX1 interaction with RCC-associated proteins G3BP1 and SPP1.

**Results:**

Knockdown of YBX1 in RCC cells dramatically inhibited cell adhesion, migration, and invasion. Mechanistic investigations revealed that YBX1 interaction with G3BP1 upregulated their downstream target SPP1 in vitro and in vivo, which led to an activated NF-κB signaling pathway. Meanwhile, knockdown of SPP1 rescued the YBX1/G3BP1-mediated activation of NF-κB signaling pathway, and RCC cell migration and invasion. We further showed that YBX1 expression was positively correlated with G3BP1 and SPP1 expression levels in clinical RCC samples.

**Conclusions:**

YBX1 interacts with G3BP1 to promote metastasis of RCC by activating the YBX1/G3BP1–SPP1–NF-κB signaling axis.

**Electronic supplementary material:**

The online version of this article (10.1186/s13046-019-1347-0) contains supplementary material, which is available to authorized users.

## Background

Renal cell carcinoma (RCC) is a deadly urological tumor that is largely incurable [1]. It accounts for approximately 90% of all primary renal neoplasms [2], and its incidence has been increasing at an alarming rate across several decades [3]. According to recent GLOBOCAN estimates, over 400,000 new cases and 175,000 deaths due to RCC were recorded worldwide in 2018 [4]. Although there are several subtypes of RCCs that differ on basis of genetics, clinical course, and therapeutic response [5], clear cell RCC (ccRCC) is the most prevalent subtype accounting the vast majority of mortality due to RCC [2]. ccRCC is characterized by increased metastases that are resistant to traditional radiotherapy and systemic therapies [6, 7]. Efforts to develop effective therapies are compromised by our limited understanding of molecular mechanisms underlying RCC invasion and metastasis. Thus, it has become imperative to identify novel molecular drivers with therapeutic potential for advanced or metastatic RCC.

Y-box binding protein 1 (YBX1) is one such molecular target that has been implicated in numerous human malignancies including breast cancer [8], prostate cancer [9], nasopharyngeal carcinoma [10], lung adenocarcinoma [11], and sarcoma [12]. YBX1 is a member of the cold-shock protein superfamily, encoded by the *YBX1* gene, that can specifically bind to the promoter of targeted genes to regulate their transcription and translation [13]. In the nucleus, YBX1 acts as a transcription factor, whereas, in the cytoplasm, it forms complexes with messenger ribonucleoproteins (mRNPs) and regulates mRNA stability [14, 15]. YBX1 is implicated in several biological processes including transcription and translation regulation, pre-mRNA splicing, DNA repair [16], stress granule formation [17], drug resistance [18], fibrogenesis [19]. In many cancers, YBX1 overexpression has been associated with poor prognosis and tumor cell proliferation [10, 11]. In RCC, although we recently showed that nuclear expression of YBX1 was correlated with T stage and metastasis [20], the underlying mechanism of YBX1 involvement in RCC metastasis remain largely unknown.

YBX1 and Ras-GTPase activating protein SH3 domain binding proteins 1 (G3BP1) were reported to exhibit highly correlated expression levels in sarcomas [17]. G3BP1 is an RNA-binding protein that possesses an acidic region, a PXXP motif and an NTF2-like domain at the N-terminus as well as two RNA-binding motifs at the C-terminus [21]. It was first identified through its ability to immunoprecipitate with the SH3 domain of Ras-GAP [22]. Previous studies showed that G3BP1 regulates mRNA stability in response to extracellular stimuli, and plays an important role in stress granule (SG) formation [23]. In addition, G3BP1 is involved in a variety of growth-related signaling pathways, such as p53 and Ras signaling [24]. Overexpression of G3BP1 has been implicated in defective signaling pathways seen several types of human tumors including gastric cancer, breast cancer, and RCC [25–27]. However, it remains poorly understood whether G3BP1 interacts with key oncoproteins such as YBX1 to modulate RCC progression and metastasis. Fully understanding the mechanisms underlying such complex interactions may unravel novel therapeutic targets for metastatic RCC.

The present study investigated the effects of YBX1 in migration, invasion, and adhesion of RCC cells both in vitro and in vivo. In addition, we characterized its interaction with two RCC-associated proteins (G3BP1 and SPP1) to decipher the functional relevance of YBX1 in RCC metastasis. Our findings indicated that YBX1 interacts with G3BP1 to promote migration and invasion of RCC cells via activating the SPP1/NF-κB signaling pathway.

## Methods

### Cell culture and transfection

The human renal cancer cell lines (786-0, ACHN and A498) and the human embryonic kidney 293 T cells were acquired from American Type Culture Collection (ATCC, USA). The ACHN and A498 cells were cultured in Eagle’s Minimum Essential Medium (MEM) (Biological Industries, Israel) while the 786-0 and 293 T cells were cultured in Dulbecco’s modified Eagle medium (DMEM) (Biological Industries, Israel), supplemented with 10% fetal bovine serum (Biological Industries, Israel) and 1% penicillin/streptomycin (BI). All cell lines were maintained at 37 °C and 5% CO_2_.

In order to generate YBX1 and G3BP1 knockdown or overexpression stable clones, 293 T cells were transfected with lentiviral vectors, including pLKO.1-Scr, pLKO.1-shYBX1, pLKO.1-shG3BP1, pWPI-Vec, and pWPI-YBX1, together with lentivirus packaging plasmids (psAX2 and pMD2G) for 48 h using Lipofectamine 2000 (Invitrogen, USA). The lentivirus supernatant was collected and then added to culture medium of RCC cells for shRNA transduction. Two days after infection, stable clones were selected with 2 μg/ml puromycin (Sangon Biotech, China) for 10 days and puromycin-resistant cells were subsequently expanded with medium containing 1 μg/ml puromycin. To generate G3BP1 overexpression cells, ACHN were then transfected with the pEGFP-C1 and pEGFP-G3BP1 constructs at 90% confluence using Lipofectamine 2000 (Invitrogen).

*SPP1*-siRNA duplexes and non-target siRNA were designed and synthesized by RiboBio (Guangzhou, China), and the sequences were listed in Table 1. For rescue experiments, ACHN-pWPI and ACHN-pWPI-YBX1; pEGFP-C1 and pEGFP-G3BP1 cells plated on six-well plates were transfected using 8 μL of the required siRNA (20 μM) together with 8 μL Lipofectamine 2000 according to the manufacturer’s instructions.

**Table 1:** The sequences of siRNAs

Name	Target sequence
si-SPP1-1	CCAGTTAAACAGGCTGATT
si-SPP1-2	GTCTCACCATTCTGATGAA
si-SPP1-3	CCAAGTAAGTCCAACGAAA

### Real-time quantitative PCR

Total RNA was isolated from RCC cells using Trizol reagent (Ambion, USA), followed by reverse transcription with FastQuant RT Kit (TIANGEN, China) according to the manufacturer’s recommendation. The reactions were performed using the ABI 7500 Fast PCR Systems (Applied Biosystems, USA). The sequences of all primers used are listed in Table 2, and GAPDH was used as the internal control.

**Table 2:** Primers sequences

Genes	Primers sequences (5′ to 3′)
*GAPDH*	F:TGCACCACCAACTGCTTAGC R:GGCATGGACTGTGGTCATGAG
*YBX1*	F:GGGTGCAGGAGAACAAGGTA R:TCTTCATTGCCGTCCTCTCT
*G3BP1*	F:GTTGCGCATTAACAGTGGTG R:CATTCAGACGGACCTCACCT
*ITGB8*	F:TGTGTGCTGGGCATGGAGAGTGT R:CAGTGCTGGGCTGCTGCTGAA
*SPP1*	F:TTTGTTGTAAAGCTGCTTTTCCTC R:GAATTGCAGTGATTTGCTTTTGC

### Western blot analysis

Cells were washed twice with ice-cold PBS, then lysed in SDS lysis buffer containing 1× protease inhibitor cocktail (Roche Applied Science, Germany). The total protein concentration in the cell lysate solution was then determined via the BCA protein assay (Thermo Fisher Scientific, USA). Protein samples (40 μg) were separated by electrophoresis on 10% SDS-PAGE and transferred to polyvinylidene difluoride membranes (Millipore, USA). After being blocked with 5% skim milk (BD Biosciences, USA) for 1 h, the membranes were incubated with specific primary antibodies; β-actin (Affinity Biosciences, USA, T0022; 1:4000), GAPDH (Immunoway Biotechnology, USA, YM3040; 1:4000), YBX1 (Santa Cruz Biotechnology, USA, sc-101,198; 1:1000), G3BP1 (Santa Cruz, sc-98,561; 1:1000), SPP1 (Abcam, USA, ab-69,498; 1:2000), p-p65 (Cell Signaling Technology, USA, #3033; 1:1000), and p65 (Cell Signaling Technology, USA, #8242; 1:1000), overnight at 4 °C. After washing with TBST, the membranes were incubated with HRP-conjugated anti-mouse (Affinity, S0002) or anti-rabbit (Affinity, S0001) secondary antibodies for 1 h and visualized with ECL system (Millipore, USA).

### Cell migration assay

Migration assay was performed using Transwell chambers (Millipore, USA) containing polycarbonate membranes with 8 μm pores. RCC cells (1 × 10^5^ cells/chamber) were added to the upper chamber in serum-free medium, and MEM medium containing 10% FBS was added to the lower chamber. After incubation for 24 h at 37 °C, the cells on the upper surface of the membrane were removed while cells attached to the lower surface of the membrane were then fixed, stained, and counted in 5 randomly chosen regions.

### Cell invasion assay

Transwell chambers were pre-coated with 50 μL mixed Matrigel (1:5 dilution; BD Biosciences, USA). RCC cells were implanted at 1 × 10^5^ cells/chamber in 200 μL serum-free medium. The lower chambers were filled with 600 μL medium supplemented with 10% FBS. After incubation for 48 h at 37 °C, the invading cells on the undersurface were fixed with 4% paraformaldehyde (Solarbio, China) and stained with hematoxylin. Cell counting was then performed using an inverted microscope.

### Cell adhesion assay

Twelve-well plates were coated with fibronectin (Sigma-Aldrich, USA) overnight at 4 °C and then washed 3 times with double distilled water (DDW). Cells were seeded at a density of 1 × 10^4^ cells/well and incubated at 37 °C for 5 min, 15 min, and 30 min. Non-adherent cells were removed by washing with PBS, and adherent cells were fixed with 4% paraformaldehyde (Solarbio, China) and stained with crystal violet.

### Microarray

Gene expression profiling was performed using Affymetrix GeneChip Microarray platform (Thermofisher, USA) to identify downstream genes regulated by YBX1. The RCC cell lines (786–0) and their corresponding YBX1 knockdown cells were sent to Jingtai Biotech company (Shanghai, China) for mRNA isolation, quality control, chip hybridization, and microarray data analysis, as previously described [13]. Briefly, cDNA was synthesized with SuperScript II (Invitrogen, USA) prior to purification with RNeasy Mini Kit (QIAGEN, USA). The samples were then labeled with biotin and hybridized at 45 °C for 16 h to GeneChip Human Gene 1.0 ST microarrays according to the manufacturer’s recommendation (Thermofisher, USA). All arrays were washed and scanned using a GeneChip Scanner 3000 (Affymetrix) at correct pixel value (3 μm) and wavelength (570 nm). After quality assessment of array data with Expression Console, high-quality data were normalized using RMA method and differential gene expression between RCC cells and corresponding YBX1 knockdown cells was performed with TAC. Genes meeting a significance threshold of FDR < 0.05 with at least 2-fold change difference were considered as differentially expressed and prioritized for further functional analysis in this study.

### Clinical specimens

Thirty-two paired fresh clear cell renal cell carcinoma (ccRCC) tissues and paired adjacent normal tissues were collected in the Second Hospital of Tianjin Medical University. The tissues were used for western blot analyses to confirm the correlation between YBX1, G3BP1, and SPP1 respectively. Patients information has been previously reported [26]. Another total of 60 formalin-fixed and paraffin-embedded primary ccRCC specimens, in which 52 specimens contained both tumor and paired adjacent normal tissues, were collected from patients in the same hospital from 2012 to 2017. These samples were used for immunohistochemistry analysis to validate results of the western blot analysis. All patients had undergone radical nephrectomy or partial nephrectomy with no preoperative and postoperative adjuvant therapies. Clinical parameters including age, gender, tumor size, histological type, and Fuhrman grade were collected. The tissue samples and clinical information were obtained with patients’ consents and ethical committee approval. According to the American Joint Commission on Cancer TNM staging system, each specimen was histologically examined, and the tumor was graded by two independent pathologists.

### Immunohistochemical staining

Formalin-fixed, paraffin-embedded RCC and paired adjacent normal tissues were cut into 5 μm thickness sections. Tissue sections were deparaffinized with xylene, dehydrated in gradient ethanol, and then antigen retrieval was performed with microwave treatment in citrate buffer (pH 6.0) (Solarbio, China). Endogenous peroxidase activity was blocked in a 15 min treatment with 3% hydrogen peroxide. The sections were then incubated with anti-YBX1, anti-G3BP1 or anti-SPP1 antibody diluted in PBS that contained 3% bovine serum albumin (Solarbio, China) overnight at 4 °C. After washing with PBS, the sections were incubated with secondary antibody (ZSGB-BIO, China) for 1 h at room temperature and visualized using the HRP DAB Detection Kit (ZSGB-BIO, China). Finally, sections were counterstained with hematoxylin.

The intensity of YBX1, G3BP1, and SPP1 immunostaining levels were evaluated independently by two pathologists based on the proportion of positively stained tumor cells (stain area) and the intensity of staining. In brief, the positive tumor cell proportion was scored as 0, 0–1% of tumor cells positive; 1, 1–5% of tumor cells were positive; 2, 6–10% of cells were positive; 3, 11–20% of cells were positive; 4, 21–50% of cells were positive; and 5, > 50% of cells were positive. The staining intensity was scored as 0, negative staining; 1, moderate staining (yellow-brown color); 2, strong staining (brown color). Then, the final staining score was calculated by combining the tumor cell proportion and staining intensity, which we recorded as: negative (0–3) and positive (4–7).

### Immunofluorescence staining

Cells were plated on poly-D-lysine coated coverslips in 12-well plates and incubated at 37 °C for 24 h. The cells were then fixed with methanol for 10 min at − 20 °C, permeabilized with 0.2% Triton X-100 in PBS for 10 min and blocked with 3% BSA in PBS for 1 h at room temperature. After incubating with the mouse monoclonal anti-YBX1 antibody (diluted 1:50) and rabbit polyclonal anti-G3BP1 antibody (diluted 1:50) overnight at 4 °C, the cells were stained with Alexa Fluor 488- and 546-conjugated secondary antibodies for 1 h at room temperature. The coverslips were then counter-stained with DAPI and observed under a confocal microscope (Olympus FV1000).

### Immunoprecipitation

Cells were harvested and lysed in the lysis buffer (40 mM Tris, 120 mM NaCl, 1% Triton X-100, 1 mM NaF, 1 mM Na_3_VO_4_) supplemented with protease inhibitor cocktail (Roche). Cell lysates were rotated for 30 min at 4 °C and pelleted by centrifugation at 4 °C, 12000 rpm for 15 min. Extracts of proteins were then incubated with indicated antibodies or control IgG for 24 h at 4 °C followed with 6 h incubation with Protein A/G Magnetic Beads (Invitrogen) at 4 °C. After a five-time wash (15 min/time) with lysis buffer, the protein samples were collected by boiling in 2× SDS loading buffer and then subjected to western blot analysis.

To further reveal the interaction site of YBX1 with G3BP1, ACHN cells were transfected with the expression vectors for GFP-tagged different domains of YBX1. The GFP-YBX1 constructs were a kind gift from the Key Laboratory of Genomic and Precision Medicine (Chinese Academy of Sciences, Beijing, China). Then the cell lysates were immunoprecipitated with GFP-Trap®_MA beads (Chromotek, Planegg-Martinsried, Germany). Beads were washed twice with lysis buffer, boiled in 2 × SDS loading buffer, and analyzed by western blot.

### Dual-luciferase reporter assay

RCC cells were seeded in triplicate in 48-well plates and grew to the proper density. Then the cells were co-transfected with 240 ng pNF-κB luciferase reporter plasmid together with 60 ng Renilla luciferase for normalization of transfection efficiency using Lipofectamine 2000 (Invitrogen). Cells were harvested after 48 h of transfection and luciferase activities were measured with the Dual-Luciferase Reporter Assay System (Promega, Madison, WI, USA) according to a protocol provided by the manufacturer. Each of the experiment was performed in triplicates.

### Xenograft tumor growth and metastasis

The BALB/c nude mice (6–8 weeks old) were randomly divided into two groups (6 mice/group). Orthotopic RCC xenograft models were established by injecting 1 × 10^6^ ACHN cells stably transfected by luciferase-labeled Scr and shG3BP1 into the sub-renal capsule of nude mice, as we described previously [26]. After eight weeks, each mice was injected with 30 μg of luciferin, and bioluminescence imaging for the detection of primary tumors and metastasis in both the liver and lung performed using the live IVIS imaging system (Perkin Elmer, USA). Then the mice were sacrificed, with their primary RCC tumors, livers, and lungs, excised and embedded in paraffin prior to sectioning. Serial 6.0 μm sections were cut and subjected to hematoxylin and eosin (H&E) staining to examine metastasis. In addition, the expression of YBX1, G3BP1, and SPP1 was detected by immunohistochemistry (IHC).

### Statistical analysis

Statistical analyses were conducted using SPSS 22.0 software (IBM, USA). All data were presented as the means ± SD. A *t-*test was performed to compare the variables of two groups, and ANOVA was used for multigroup comparisons. Chi-square test was used for statistical analysis of the correlations between protein expression and clinicopathological features. The correlations between the expressions of YBX1, G3BP1, and SPP1 were analyzed using Spearman test. Differences with *p < 0.05 were considered as statistically significant.

## Results

### YBX1 is critical for RCC cell adhesion, migration, and invasion

**Fig. 1:**
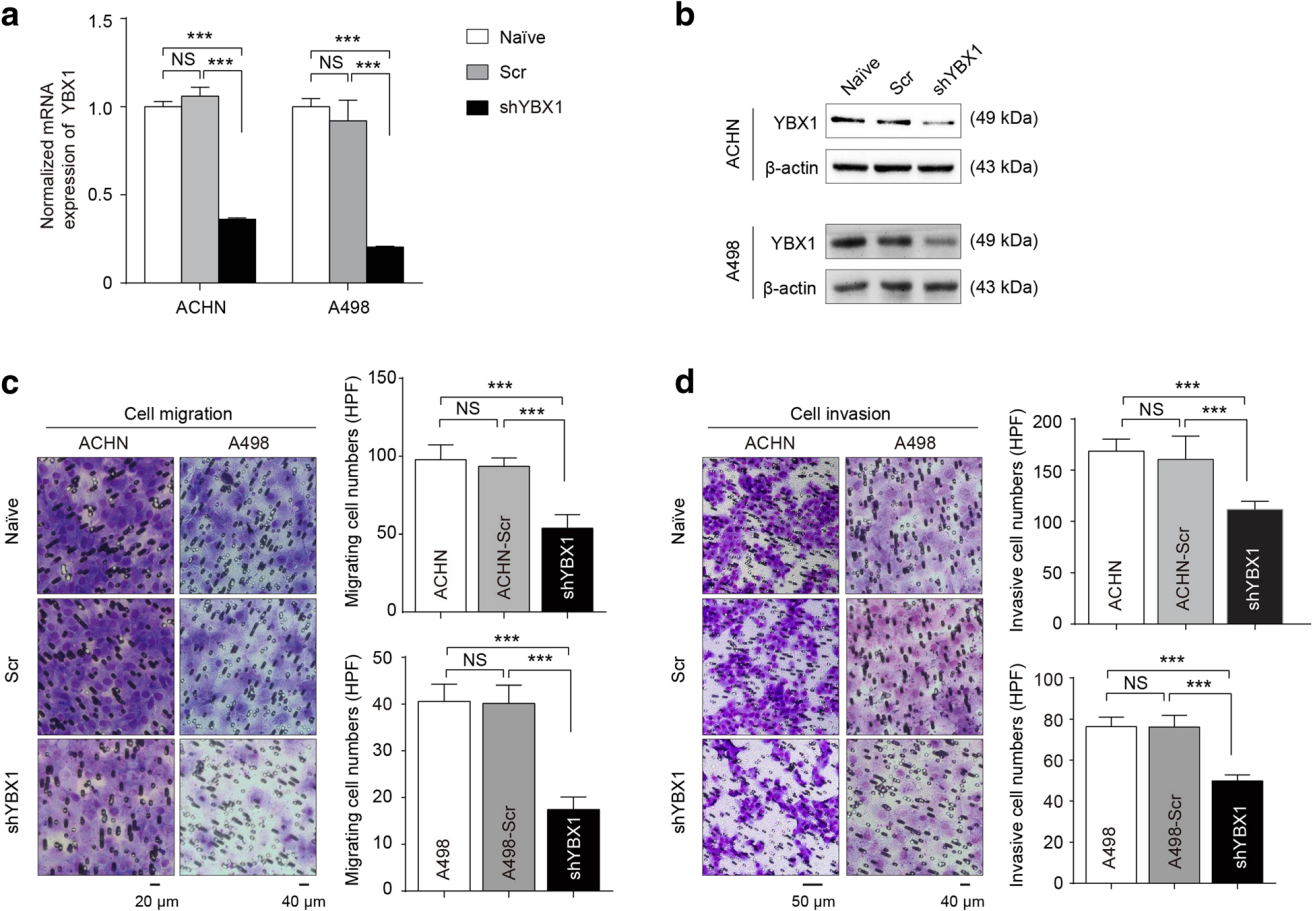
YBX1 is critical for RCC cell migration, invasion, and adhesion. The expression of YBX1 was detected after lentivirus transfection by real-time PCR (**a**) and western blot (**b**). β-actin was used as loading control, respectively. ACHN and A498 cells stably expressing shYBX1 were subjected to Migration (**c**) and Matrigel-coated invasion assays (**d**). Left panels: the representative images of migrated/invaded cells, Naïve: untreated; Scr: scramble control; shYBX1: YBX1 knockdown. Right panels: histograms, corresponding to left panels, show means ± SD of cell numbers from three independent assays. Statistically significant differences were indicated: ***, *P* < 0.001. NS: no significant difference

Our previous study demonstrated that nuclear levels of YBX1 were associated with T stage and metastasis of RCC, which prompted us to explore the functional effects of YBX1 [20, 28]. YBX1 stable knockdown RCC cell lines ACHN and A498 were established via lentiviral infection. The efficiency of YBX1 knockdown in these two cell lines was determined by real-time PCR and western blot (Fig. 1a and b), respectively. YBX1 knockdown cells expressing < 35% of detectable YBX1 in comparison to the scramble control cells were qualified as YBX1 knockdown and used for further experiments. YBX1 knockdown ACHN and A498 cells were subjected to adhesion, transwell migration, and invasion assays. Tumor cell adhesion to extracellular matrix and basement membranes has been considered as a crucial step in metastasis [29]. Thus, we examined the influence of YBX1 on the adhesion of RCC cells to the substrates pre-coated with fibronectin, which is a major basement membrane component [29]. As shown in Fig. 1c, downregulation of YBX1 significantly (*p* < 0.01) inhibited migration in both ACHN and A498 cells. Meanwhile, YBX1 downregulation led to a decreased number of RCC cells passing through the Matrigel-coated membrane (Fig. 1d). Further cell adhesion assay showed that depletion of YBX1 significantly inhibited RCC cell adhesion to fibronectin (Additional file 1: Figure S1A and S1B). Together, these findings suggested that aberrant expression of YBX1 was involved in metastatic phenotypes of RCC cells.

### The interaction between YBX1 and G3BP1 in RCC

**Fig. 2:**
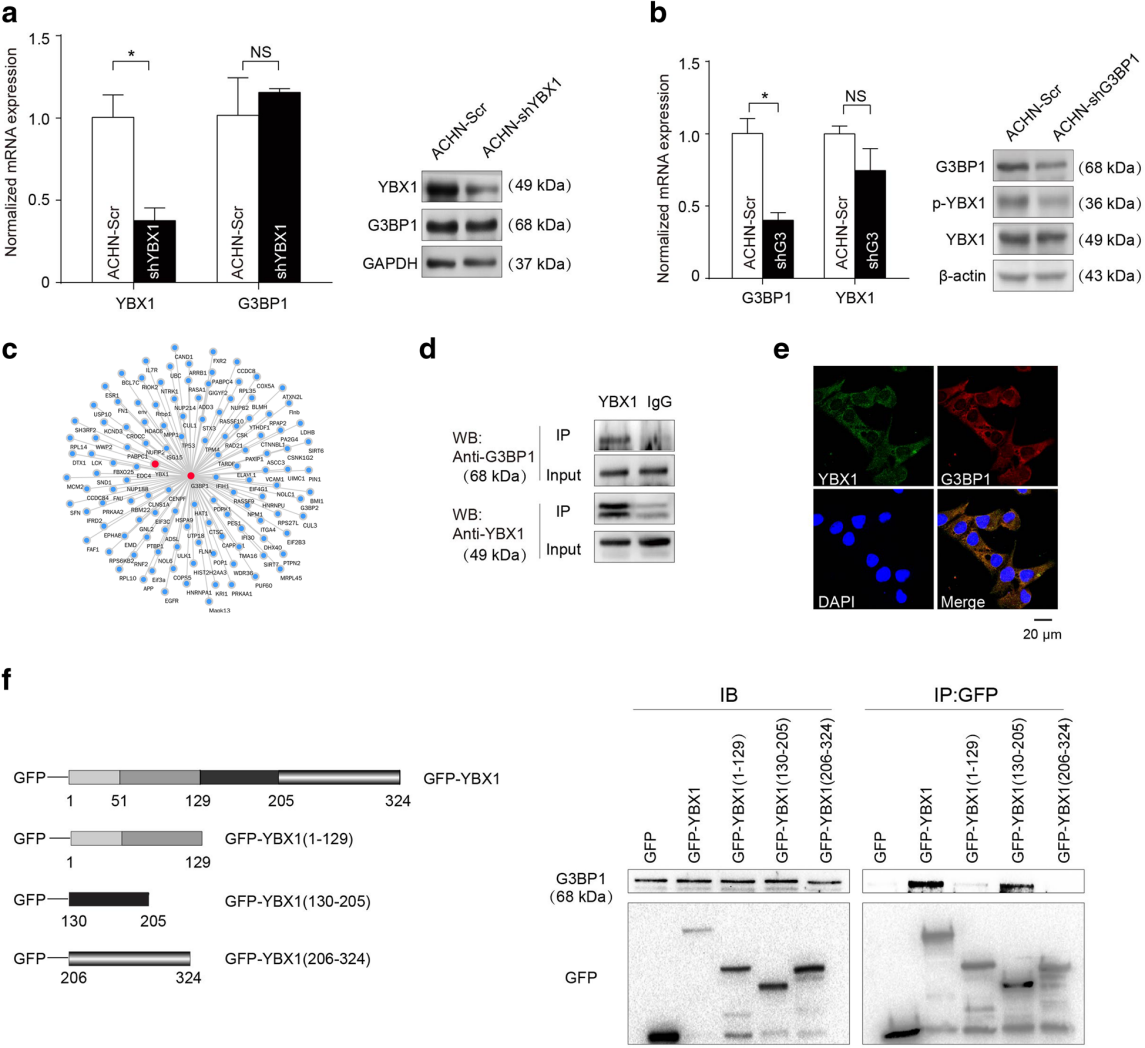
The interaction between YBX1 and G3BP1 in RCC. (**a**) Left panels: YBX1 and G3BP1 mRNA levels were quantified by real-time PCR with YBX1 knockdown in ACHN cells. Right panels: the expression of YBX1 and G3BP1 in YBX1-depleted ACHN cells were detected by western blot. (**b**) G3BP1 did not affect YBX1 mRNA and protein level in ACHN cells knockdown G3BP1. (**c**) The interaction network of G3BP1 protein with other proteins was identified using STRING database. Abbreviations: STRING, Search Tool for the Retrieval of Interacting Genes. (**d**) ACHN cell lysates were mixed with an anti-YBX1 antibody or IgG antibody that does not recognize YBX1. YBX1 and control immunoprecipitates were analyzed by western blot with anti-G3BP1 and anti-YBX1 antibodies. IP, immunoprecipitation. (**e**) Immunofluorescence assays in ACHN cells. The localizations of YBX1 and G3BP1 were detected by confocal laser scanning microscopy as indicated. Scale bar = 20 μm. (**f**) Left panels: schematic diagram of YBX1 domain. Right panels: Co-IP assay showing interaction of G3BP1 with YBX1 (aa 130–205). Statistically significant differences were indicated: *, *P* < 0.05. NS: no significant difference

We next investigated the underlying molecular mechanisms through which YBX1 regulates RCC cells migration and invasion. With evidence from our recent study showing that G3BP1 contributes to the metastasis of RCC in both in vitro assay and in vivo xenograft model [26], and others indicating that YBX1 and G3BP1 expression levels are highly correlated in human sarcomas [17], we examined whether YBX1 affects G3BP1 expression and cellular localization in RCC. However, the results indicated that depletion of YBX1 had no effect on G3BP1 mRNA and protein levels (Fig. 2a). Similarly, no effect on YBX1 expression was observed following depletion of G3BP1 (Fig. 2b). The STRING 10.5 database [30], was then queried to predict G3BP1-interacting proteins. As shown in Fig. 2c, YBX1 interacted directly with G3BP1. To validate this interaction, total proteins from ACHN cells were extracted and co-immunoprecipitation (co-IP) experiments were performed with antibodies detecting the endogenous proteins. The presence of G3BP1 in the endogenous YBX1 complex confirmed the interaction between YBX1 and G3BP1 (Fig. 2d). Meanwhile, immunofluorescent staining showed the colocalization of endogenous YBX1 (green) and G3BP1 (Red) in RCC ACHN cell cytoplasm (Fig. 2e).

To further determine the specificity of the interaction, we took advantage of a series of YBX1 mutants that contained N-terminal domain and cold shock domain (aa 1–129), and part of C-terminal domain (aa 130–205 or aa 206–324) (Fig. 2f, top panel). ACHN cells were transfected with GFP-YBX1 or each of the GFP-YBX1 mutants. YBX1 and its mutants were expressed at the expected molecular weights (Fig. 2f, bottom panel). Immunoprecipitation with an anti-GFP antibody, followed by western blot analysis using anti-G3BP1 antibody showed that deletion of the C-terminal domain of YBX1 (aa 130–205) prevented its complex formation with G3BP1 (Fig. 2f, bottom panel). Taken together, these results further confirmed the complex formation between YBX1 and G3BP1 and suggest that the YBX1 C-terminal domains (aa 130–205) are directly involved in the complex formation.

Collectively, these results indicated that YBX1 specifically interacted with G3BP1 in RCC cells.

### YBX1/G3BP1 complex activate downstream NF-κB signaling pathway via up-regulating SPP1 in RCC cells

To further delineate the underlying molecular mechanisms through which YBX1 regulates cell metastatic abilities, the downstream genes regulated by YBX1 were identified via microarray gene expression profiling and their functional relevance was characterized using in silico approaches. Indeed, the analysis revealed that genes encoding molecules involved in enriched pathways such as cell adhesion, ECM-receptor interaction and sphingolipid metabolism were significantly down-regulated after YBX1 knockdown (Additional file 2: Figure S2A). Among these, *ITGB8*, *RELN,* and *SPP1* were the top tumor-promoting candidates significantly downregulated (*FDR* < 0.05) by YBX1 knockdown in the ECM-receptor interaction pathway (Table 3). Further gene set enrichment analysis (GSEA) confirmed *SPP1* involvement in the EMT process regulated by YBX1 (Additional file 2: Figure S2B and S2C). Because SPP1 is frequently overexpressed in multiple cancers [31, 32], is associated with defective apoptosis and invasion in RCC cells [33], and was dramatically downregulated after YBX1 knockdown, we prioritized SPP1 for further investigation.

**Table 3:** The differentially expressed genes were enriched in ECM-receptor interaction pathway after YBX1 knockdown

Gene name	Fold change	FDR
*ITGB8*	-5.47	2.58E-06
*RELN*	-2.20	3.57E-05
*SPP1*	-2.93	4.10E-05

**Fig. 3:**
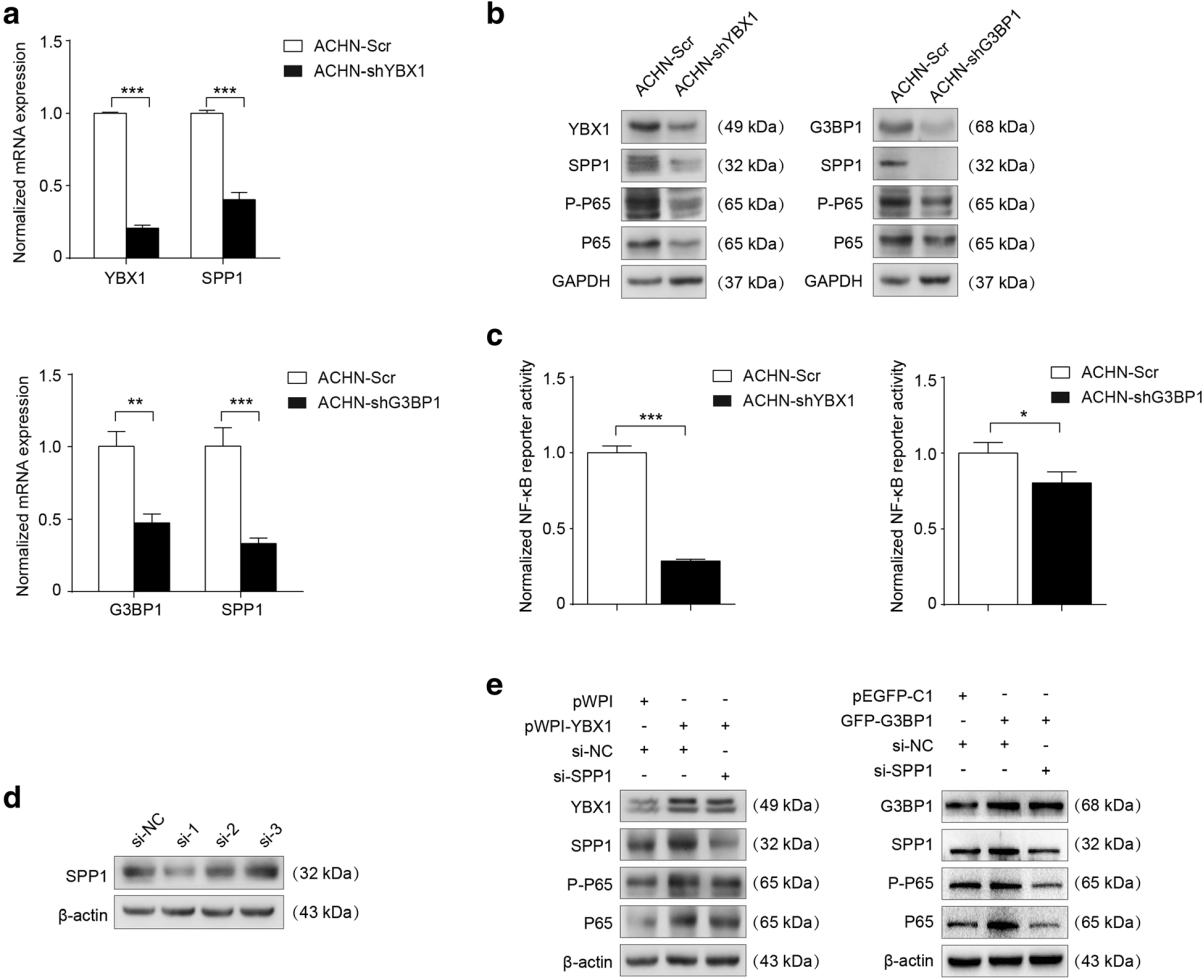
YBX1/G3BP1 complex up-regulates SPP1 to activate downstream NF-κB signaling pathway in RCC cells. (**a**) Analysis of the effect of YBX1 knockdown (upper panel) or G3BP1 knockdown (lower panel) on *SPP1* mRNA levels using real-time PCR. (**b**) Western blot assays were performed to measure the YBX1, G3BP1, SPP1, p-p65 (Ser536), and total p65 in treated ACHN cells. GAPDH was used as an internal control. (**c**) Cells were co-transfected with NF-κB pathway firefly luciferase reporter together with internal control Renilla luciferase reporter (pRL-TK) vector. Then, luciferase activity was measured using a dual-Luciferase Reporter Assay System (Promega). (**d**) SPP1 knockdown by using three independent *SPP1* siRNAs (*si-SPP1–1, si-SPP1–2, si-SPP1–3*) in ACHN cells were evidenced by western blot. β-actin was used as loading control. ACHN cells were transfected with pWPI+si-NC, pWPI-YBX1 + si-NC, pWPI-YBX1 + si-SPP1; pEGFP-C1 + si-NC, GFP-G3BP1 + si-NC, GFP-G3BP1 + si-SPP1, and then (**e**) the expression levels of YBX1, G3BP1, SPP1, p-p65 (Ser536), and total p65 were examined by western blot. β-actin was used as an internal control. Data were presented as mean ± SD. Statistically significant differences were indicated: *, *P* < 0.05; **, *P* < 0.01; ***, *P* < 0.001

Consistent with the microarray data, YBX1 knockdown decreased the expression of the *SPP1* mRNA (Fig. 3a, upper panel; Additional file 2: Figure S2D). Further western blot results confirmed that depletion of YBX1 also inhibited the protein level of SPP1 (Fig. 3b, left panel; Additional file 2: Figure S2E). To explore the underlying molecular mechanism of YBX1 interaction with G3BP1 in RCC progression, we investigated the effects of G3BP1 on the oncogenic signaling pathways that can be affected by YBX1 silencing. Our data showed that the expression of SPP1 in both mRNA and protein levels were down-regulated in G3BP1 knockdown RCC cells, suggesting a functional role of the YBX1/G3BP1 complex in the regulation of SPP1 (Fig. 3a, lower panel; Fig. 3b, right panel). SPP1 was reported as a direct regulator of NF-κB signaling pathway [34]. In line with this notion, we examined the effects of YBX1 on the NF-κB protein levels. As shown in Fig. 3b and Fig. 3c, YBX1 knockdown inhibited the phosphorylation of NF-κB subunit p65 (Ser536) together with the total amount of p65 protein levels in RCC cells. Similar results were obtained in G3BP1 knockdown ACHN cells (Fig. 3b, right panel). In addition, the effects of YBX1 and G3BP1 on NF-κB signaling pathway were examined by dual-luciferase reporter assay. The results indicated that NF-κB reporter activity was not only significantly decreased by YBX1 knockdown but also G3BP1 depletion (Fig. 3c; Additional file 2: Figure S2F).

In order to determine whether YBX1/G3BP1 complex could regulate downstream NF-κB signaling pathway via SPP1 in RCC cells, we first selected the most effective siRNAs against *SPP1* (*si-SPP1–1, si-SPP1–2, si-SPP1–3*) in ACHN cells (Fig. 3d). Then, the ACHN cells were transfected with pWPI+si-NC, pWPI-YBX1 + si-NC, pWPI-YBX1 + si-SPP1; pEGFP-C1 + si-NC, GFP-G3BP1 + si-NC, GFP-G3BP1 + si-SPP1. The transfection efficiency and the effect of the YBX1/G3BP1 regulatory axis on its downstream signaling pathways were verified by western blot. The results revealed that downregulation of SPP1 expression abolished YBX1/G3BP1-induced NF-κB expression, suggesting that SPP1 was required for the YBX1/G3BP1-induced activation of NF-κB signaling (Fig. 3e).

Thus, these results suggested that YBX1 interaction with G3BP1 up-regulates SPP1 to activate NF-κB signaling pathway.

### YBX1/G3BP1 regulated the migration and invasion abilities of RCC cells via SPP1

**Fig. 4:**
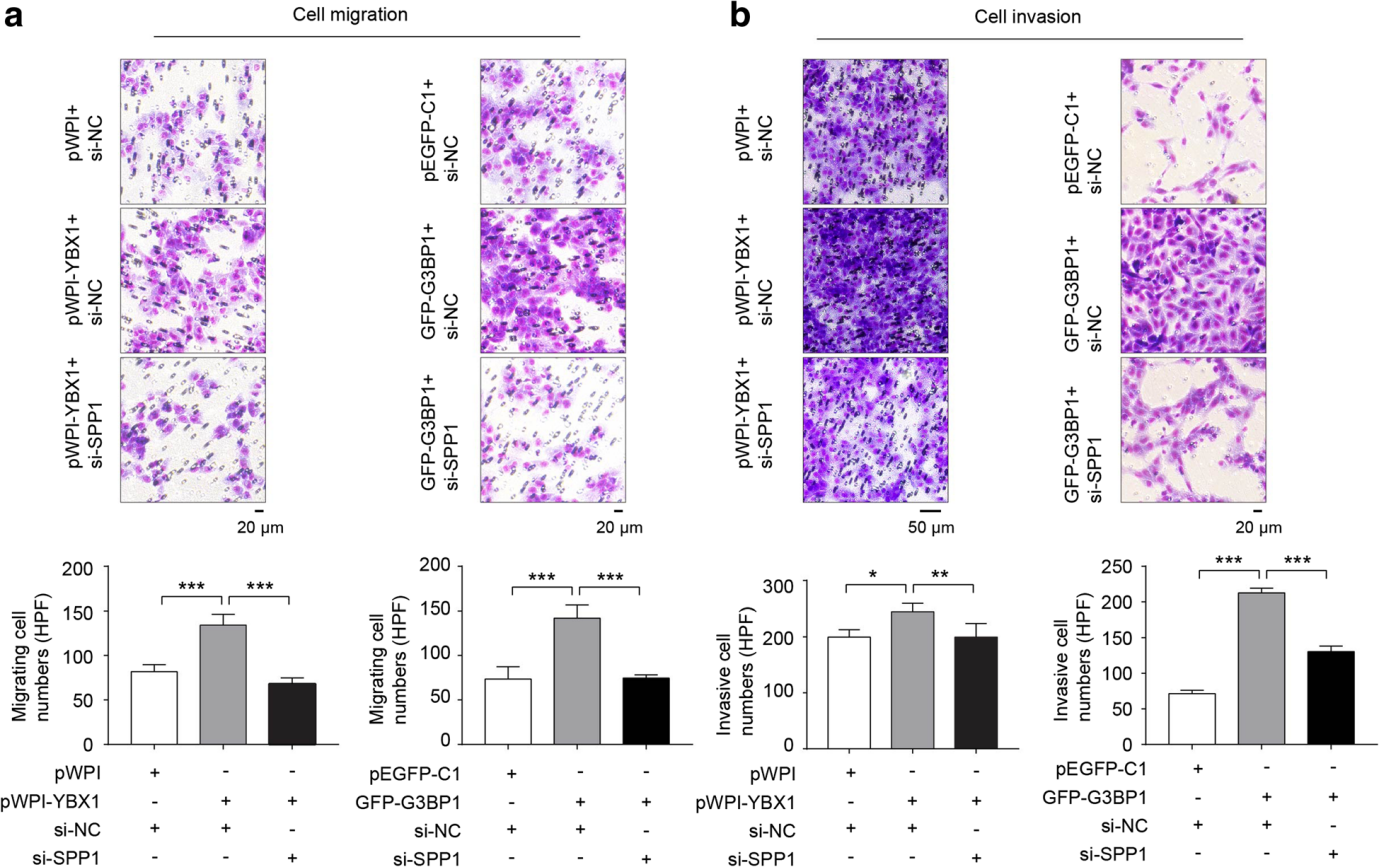
YBX1/G3BP1 affects the migration and invasion abilities of RCC cells via SPP1. (**a**) Transwell assay was performed to determine the cell migration in treated ACHN cells. (**b**) Cell invasive assay was used to examine the cell invasion in treated ACHN cells. Upper panel: representative microscopic images. Lower panel: quantitative analysis. Data were presented as mean ± SD from three independent experiments, and five random microscopic fields were acquired in each experiment for quantification. Statistically significant differences were indicated: *, *P* < 0.05; **, *P* < 0.01; ***, *P* < 0.001

To determine whether YBX1/G3BP1 complex was involved in RCC cells metastasis through regulating SPP1, we verified the role of SPP1 on YBX1 or G3BP1 regulated cell migration and invasion in ACHN cells. The results showed that YBX1 or G3BP1 overexpression significantly (*p* < 0.01) promoted tumor cell migration (Fig. 4a and b) and invasion in RCC ACHN cells, while SPP1 depletion strongly attenuated the effect of YBX1 or G3BP1 induced RCC cells migration and invasion (Fig. 4a and b). Based on these results, YBX1/G3BP1 increased RCC cell migration and invasion, which could potentially be declined by the knockdown of SPP1.

### Expression of YBX1 was correlated with G3BP1 and SPP1 in RCC patients

**Fig. 5:**
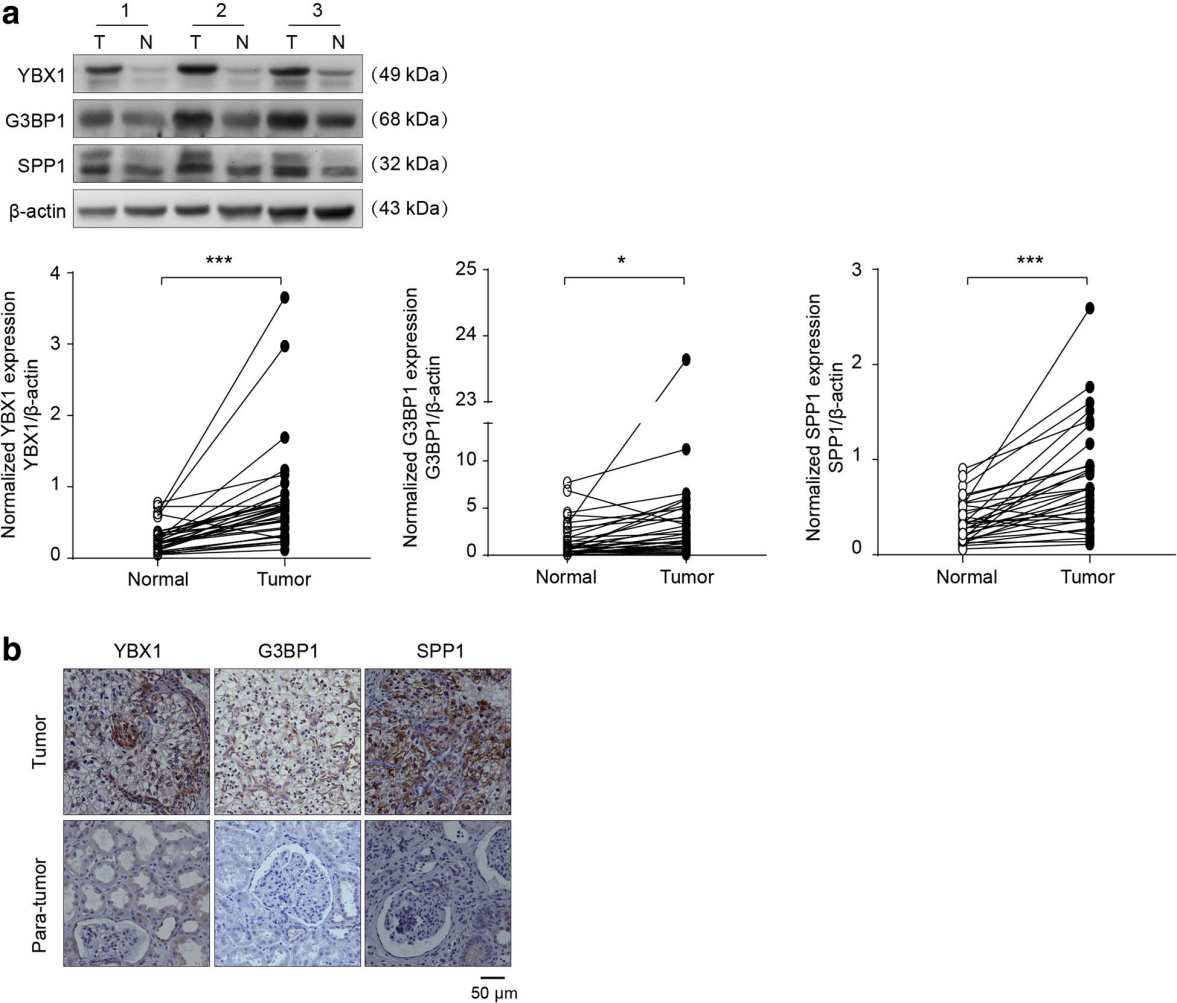
Clinical relevance of YBX1, G3BP1 and SPP1 in primary RCC patients. (**a**) The expressions of YBX1, G3BP1, and SPP1 in 32 freshly collected clinical primary RCC (T) and paired adjacent normal kidney tissues (N). Upper panel: representative three pairs of western blot images. Lower panel: the expressions of YBX1, G3BP1, and SPP1 was quantified by normalizing with β-actin. (**b**) YBX1 levels were positively associated with G3BP1 and SPP1 expression in 60 primary RCC specimens. Representative cases are shown. Scale bar = 50 μm. Statistically significant differences were indicated: *, *P* < 0.05; ***, *P* < 0.001

To confirm the findings implicating YBX1/G3BP1–SPP1 regulatory axis in RCC migration and invasion, we next investigated the correlations of YBX1 with G3BP1 and SPP1 using 32 freshly collected clinical RCC and paired adjacent normal kidney tissues. Western blot revealed that the expressions of YBX1, G3BP1, and SPP1 were significantly (*p* < 0.05) higher in RCC tissues compared to the corresponding adjacent normal kidneys (Fig. 5a). In addition, YBX1 protein levels were positively correlated with G3BP1 (*r* = 0.537, *p* = 0.002) and SPP1 (*r* = 0.803, *p* < 0.0001) expression levels (Fig. 5a; Table 4). Consistent with western blot results, IHC analysis of 60 tissue specimens also showed that YBX1 expression was positively correlated with G3BP1 (*r* = 0.483, *p* < 0.0001) and SPP1 (*r* = 0.571, *p* < 0.0001) expression (Fig. 5b; Table 5). At the same time, we analyzed the association among YBX1, G3BP1 and SPP1 by the LinkedOmics database and found that YBX1 was positively correlated with G3BP1 (R = 0.55, *p* < 0.05). G3BP1 was positively correlated with SPP1 (R = 0.61, *p* < 0.05) (Additional file 3: Figure S3). Additionally, we investigated the association of YBX1, G3BP1 and SPP1 expression with several clinical parameters including gender, age, tumor size, TNM stage and Fuhrman grade (Additional file 5: Table S1). The results revealed that the expression levels of YBX1, G3BP1, and SPP1 in RCC patients were significantly associated with elevated levels of TNM stages and Fuhrman grade. In summary, our results indicated that the expression of YBX1, G3BP1, and SPP1 is increased in RCC and its expression level is positively correlated with G3BP1 and SPP1 in RCC tissues.

**Table 4:** Clinical relevance of YBX1, G3BP1 and SPP1 in RCC by western blot

	YBX1 expression		Spearman’s *r* Correlation coefficient	*p* Value
	Up	Down		
G3BP1				
Up	26	0	0.537	0.002
Down	4	2		
SPP1				
Up	29	0	0.803	<0.0001
Down	1	2		

Note: The expressions of YBX1, G3BP1 and SPP1 were quantified and comparedbetween RCC tumor (T) and paired adjacent normal tissue (N). The relative ratioT/N > 1 was defined as “Up”, and T/N < 1 was defined as “Down”

**Table 5:** Clinical relevance of YBX1, G3BP1 and SPP1 in RCC by IHC

	YBX1 expression		Spearman’s *r* Correlation coefficient	*p* Value
	+	-		
G3BP1				
+	33	5	0.483	<0.0001
-	9	13		
SPP1				
+	36	5	0.571	<0.0001
-	6	13		

### The interaction between YBX1 and G3BP1 in nude mice

**Fig. 6:**
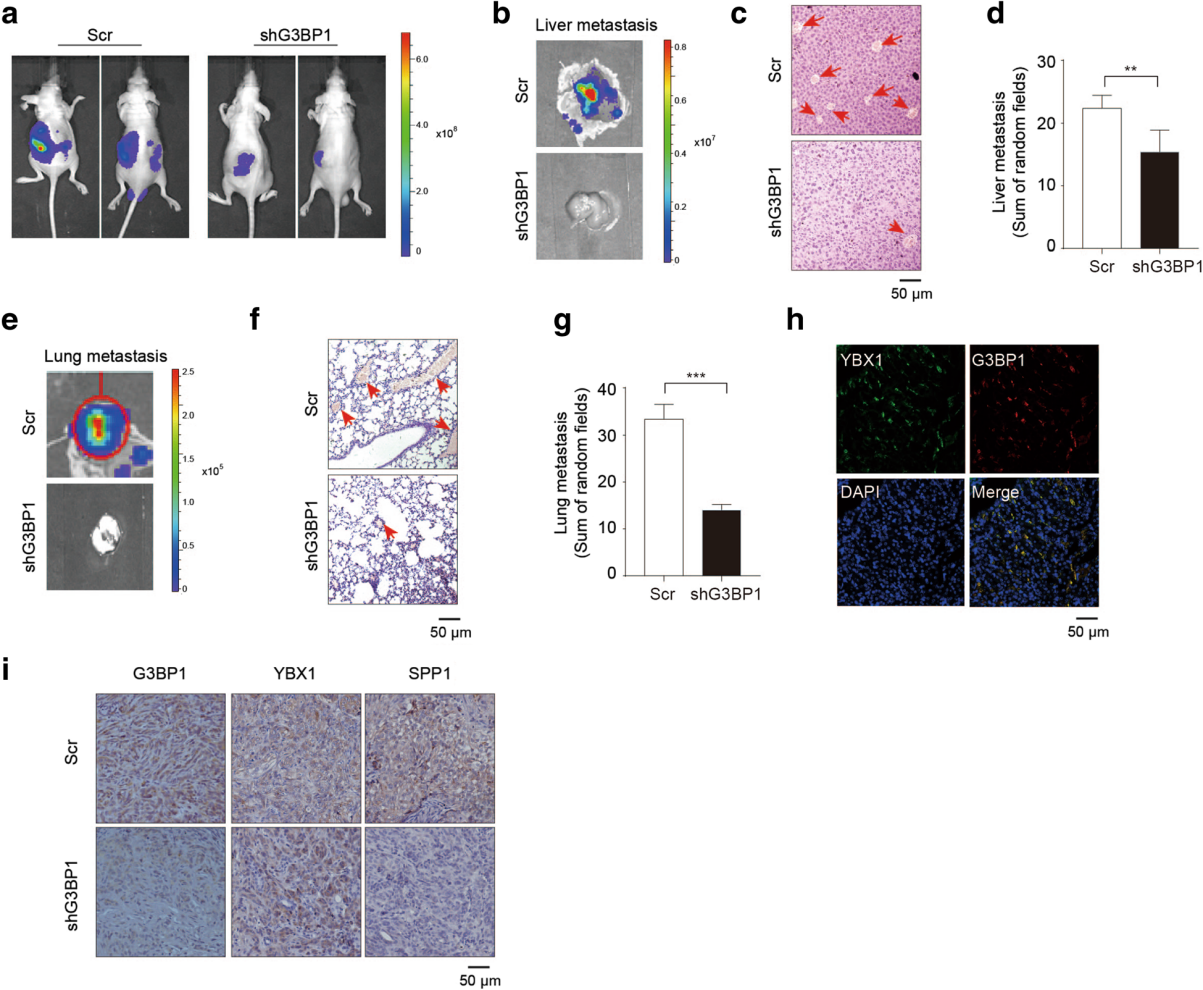
The interaction between YBX1 and G3BP1 in nude mice. (**a**). ACHN-Scr cells and ACHN-shG3BP1 cells were labeled with luciferase gene, and 1 × 10^6^ cells were injected into the sub-renal capsule. Tumor growth was monitored by IVIS imaging system, and representative bioluminescence images were shown. Liver (**b**) and lung (**e**) metastases were examined by IVIS bioluminescence imaging system, and representative images were shown. Representative H&E staining of Liver (**c**) and lung (**f**) isolated from orthotopic xenograft models to detect metastatic foci. Tumor metastatic foci in liver (**d**) and lung (**g**) were quantified and compared using five random views under the microscope. (**h**) Co-localization of YBX1 and G3BP1 was examined by Immunofluorescence in xenograft model. (**i**) Representative tumor sections from G3BP1 knockdown and control mice were subjected to immunohistochemistry staining using the YBX1, G3BP1 and SPP1 antibodies. Scale bar = 50 μm. Statistically significant differences were indicated: **, *P* < 0.01; ***, *P* < 0.001

To further confirm the functional correlation between YBX1 and G3BP1 in vivo, we performed xenograft tumor experiments, as we described previously [26]. Interestingly, bioluminescent signals indicated that mice implanted with G3BP1 knockdown group had much smaller tumor and fewer liver and lung metastatic foci detected than those in scramble controls. Further H&E staining of the excised livers and lungs clearly demonstrated that significantly (*p* < 0.01) more metastatic nodules were found in the scramble control group than in the G3BP1 knockdown group (Additional file 4: Figure S4; Fig. 6 a-g).

Immunofluorescence was conducted to confirm YBX1 and G3BP1 co-localization in the cytoplasm in xenograft mouse models (Fig. 6h). In addition, immunohistochemistry staining of the xenograft renal tissues confirmed that YBX1 expression remained unchanged in the G3BP1 knockdown tumors compared to the control tumors, while the expression of SPP1 was significantly decreased in the G3BP1 knockdown tumors when compared to the control tumors (Fig. 6i).

Collectively, the results from the in vivo tumor xenograft models indicated that silencing of G3BP1 suppresses RCC tumor cell metastasis through YBX1/G3BP1-SPP1 signaling pathway.

## Discussion

Metastatic RCC is still largely incurable despite recent therapeutic advances [6, 7]. Efforts to identify novel molecular drivers with therapeutic potential are compromised by our poor understanding of the molecular mechanism underlying RCC invasion and metastasis. In the present study, we found that YBX1 and G3BP1 regulated RCC cell migration and invasion. Consistently, our in vivo orthotopic tumor xenografts results confirmed that knockdown of G3BP1 suppressed RCC tumor metastasis in mice. We revealed that the expression levels of YBX1, G3BP1, and SPP1 were increased in primary RCC as compared to adjacent normal tissues and were associated with higher levels of TNM stages and Fuhrman grade. Remarkably, we demonstrated that YBX1 interacts with G3BP1 to upregulate SPP1 and activate NF-κB, which eventually promotes RCC metastasis. Together, our findings indicate that the interaction of YBX1 with G3BP1 promotes RCC metastasis through YBX1/G3BP1-SPP1-NF-κB signaling axis.

YBX1 has been associated with tumorigenesis, tumor progression and resistance to chemotherapy in many cancers [35–39]. However, there is still limited knowledge about its role in the progression of RCC. YBX1 knockdown has been shown to inhibit lung adenocarcinoma cells growth [38]. Johnson et al. reported that YBX1 knockdown was associated with decreased malignant pleural mesothelioma (MPM) cell proliferation, colony formation, migration, and invasion [39]. In addition, overexpression of YBX1 was reported to enhance the metastatic potential of osteosarcoma cells by inducing EMT and migration [37]. Previously, we showed that YBX1 expression is significantly elevated in clinical RCC patients in comparison to paired adjacent normal kidney tissues. Moreover, we found the nuclear expression levels of YBX1 in RCC tissues were correlated with T stage and tumor metastasis [13, 20]. Consistently, we demonstrated that knockdown of YBX1 significantly inhibited the adhesion, migration and invasion abilities of RCC cells in this present study, indicating that YBX1 promotes RCC progression.

Somasekharan et al. found YBX1 affected G3BP1 protein synthesis by directly binding to the 5′ untranslated region of *G3BP1* mRNAs [17]. Our previous studies have identified a total of 129 proteins including G3BP2, sharing ~ 70% sequence similarity with G3BP1 [40], that potentially interact with YBX1 [20]. Also, we demonstrated that the expression of G3BP1 was increased in RCC compared to paired adjacent normal kidney tissues and was correlated with RCC progression. Moreover, the knockdown of G3BP1 inhibited RCC cell proliferation, migration, and invasion in vitro and in vivo [26]. In this study, immunoprecipitation revealed that YBX1 could interact with G3BP1 in RCC cells and their co-localization in the cytoplasm was validated by immunofluorescence in vitro using RCC cells and in vivo using Xenograft models. YBX-1 protein has three independent domains; an amino-terminal region (N-terminal domain, aa 1–50), a cold shock domain (aa 51–129) and a carboxy-tail region (C-terminal domain, aa 130–324). The central cold shock domain is a highly conserved nucleic acid recognition domain, containing RNP-like motifs. The C-terminal domain consists of alternating positively and negatively charged regions, called charged zipper domains, and is thought to interact with other cellular proteins or nucleic acids [15]. The present study demonstrates that YBX1 can directly bind to G3BP1 through its C-terminal domain (aa 130–205). This region contains a part of the charged zipper which consists of highly acidic and basic charged areas. Taken together, YBX1 could interact with G3BP1 to control RCC metastasis.

The secreted phosphoprotein 1 (SPP1, also known as osteopontin), is an extracellular matrix chemokine-like phosphoglycoprotein that facilitates cell-matrix interaction and promotes cancer progression [41]. Matusan and colleagues found high levels of SPP1 in ccRCC was significantly correlated with tumor size, Fuhrman nuclear grade, pathological stage, and Ki-67 proliferation index [42]. Recent studies also revealed that higher SPP1 expression was significantly correlated with a shortened overall survival in ccRCC [43]. Remarkably, our results showed that YBX1 and G3BP1 regulated SPP1 expression. We demonstrated that knockdown of YBX1 or G3BP1 reduced SPP1 expression at both mRNA and protein levels. This notion was further supported by detecting the expression of YBX1, G3BP1, and SPP1 in clinical RCC tissue samples, which revealed the positive correlations of YBX1, G3BP1, and SPP1. Meanwhile, high levels of YBX1, G3BP1, and SPP1 in RCC are correlated with TNM stage and Fuhrman grade. Functionally, our results demonstrated that SPP1 depletion strongly attenuated the effect of YBX1 or G3BP1 induced RCC cells migration and invasion. This perhaps implies that YBX1/G3BP1 functions through SPP1 signaling and eventually contributes to RCC metastasis.

Nuclear factor κB (NF-κB) is a transcription factor that regulates cell proliferation, apoptosis, epithelial-mesenchymal transition (EMT) and chemotherapy resistance in various cancer cells [44–47]. NF-κB maintains an inactive form in the cytoplasm by binding to inhibitors of kappa B proteins (IκB). In response to various stimulation, IκB kinase (IKK) is activated and phosphorylates IκB. Then the proteasome-mediated degradation of IκB exposes the nuclear localization signal of NF-κB, thus allowing its translocation to the nucleus where it activates the transcription of various target genes [47]. Pei et al. found that inhibition of NF-κB pathway attenuates cell migration ability in ccRCC cells [45]. SPP1 have been demonstrated to induce NF-κB activation in breast cancer cells [48]. In a recent study, YBX1 was also found to exert important activities in the NF-κB pathway in human neuroblastoma cells [49]. Based on these findings, we evaluated the effect of YBX1 on aberrant NF-κB signaling in RCC cells. Our results showed that knockdown YBX1 significantly inhibited phosphorylation of p65 (Ser536) together with the total amount of p65 protein levels in RCC cells. In addition, we demonstrated that depletion of SPP1 with siRNA significantly decreased the levels of both phosphorylated and total p65 in YBX1 overexpression RCC cells. We also found that knockdown of SPP1 reversed the promoting effect of G3BP1 on NF-κB activation.

Further, ccRCCs are characterized by inactivation of the von Hippel–Lindau tumor suppressor (VHL), which is lost in up to 90% of ccRCCs [50, 51]. VHL loss can also lead to the activation of NF-κB, which is associated with ccRCC progression [52, 53]. Thus, we examined the effects of YBX1 and G3BP1 in regulating RCC progression using both the VHL mutant (A498) and VHL wild-type (ACHN) RCC cell lines. Our observation of similar results using both cell lines suggests, therefore, that YBX1 and G3BP1 promote RCC metastasis independent of VHL. However, the exact mechanism of this YBX1 and G3BP1-induced NF-κB signaling pathway remains to be determined.

## Conclusions

We have demonstrated that YBX1 and G3BP1 show a significant promoting effect on migration, invasion, and adhesion of RCC cells. Mechanistically, YBX1 interaction with G3BP1 results in SPP1 enrichment and NF-κB activation, which promotes migration and invasion of RCC cells.

## Additional files

Additional file 1: **Figure S1.** YBX1 promotes the adhesion ability of RCC cells. (A) Depletion of YBX1 decreased ACHN cells adhesion ability at 5 min, 15 min and 30 min. (B) Depletion of YBX1 significantly decreased A498 cells adhesion ability at 5 min, 15 min. Upper panel: representative microscopic images. Lower panel: quantitative analysis. The data were presented as mean ± SD of three independent experiments, and five random microscopic fields were acquired in each experiment for quantification. Statistically significant differences were indicated: **, *p* < 0.01; ***, *P* < 0.001. NS: no significant difference. (JPG 242 kb)

Additional file 2: **Figure S2.** YBX1 regulates SPP1 to activate downstream NF-κB signaling pathway in RCC cells. (A) The genes regulated by YBX1 were enriched in sphingolipid metabolism, axon guidance, chemical carcinogenesis, cell adhesion molecules (CAMs), ECM-receptor interaction, tryptophan metabolism pathways. (B) Gene set enrichment analysis was performed to identify genes that have positive or negative correlations with YBX1 expression. Enrichment plots showed significant correlation of the EMT process. (C) Heat maps for genes upregulated or downregulated by YBX1 knockdown in EMT process. (D) YBX1 knockdown decreased the level of the SPP1 mRNA in 786–0 cells. (E) The expressions of YBX1, SPP1, p-p65 (Ser536), and total p65 were examined by western blot in A498-Scr and A498-shYBX1 cells. (F) 786–0 cells stably knockdown YBX1 and control cells were transiently with NF-κB pathway firefly luciferase reporter together with internal control Renilla luciferase reporter (pRL-TK) vector. Then, luciferase activity was measured using a dual-Luciferase Reporter Assay System (Promega). Statistically significant differences were indicated: **, *p* < 0.01; ***, *P* < 0.001. NS: no significant difference. (JPG 241 kb)

Additional file 3: **Figure S3.** Correlation between the expression of YBX1, G3BP1 and SPP1 from LinkedOmics database. (A) YBX1 was positively correlated with G3BP1. (B) G3BP1 was positively correlated with SPP1. (JPG 28 kb)

Additional file 4: **Figure S4.** The bioluminescence intensity of RCC in G3BP1 knockdown group and control group in vivo. (JPG 11 kb)

Additional file 5: **Table S1.** Expression of YBX1, G3BP1 and SPP1 in RCC tissues. (DOCX 13 kb)

## Data Availability

The datasets used and/or analysed during the current study are available from the corresponding author on reasonable request.

## Abbreviations

ccRCC: Clear cell renal cell carcinoma
cDNA: Complementary DNA
CO_2_: Carbon dioxide
DDW: double distilled water
ECM: Extracellular matrix
FDR: False discovery rate
G3BP1: Ras-GTPase activating protein SH3 domain binding proteins 1
GSEA: Gene set enrichment analysis
IHC: Immunohistochemistry
ITGB8: Integrin Subunit Beta 8
NaCl: Sodium chloride
NF-κB: Nuclear Factor kappa B
NTF2: nuclear transport factor 2
PCR: Polymerase chain reaction
PxxP: Proline-rich (*PxxP*) motifs
RCC: Renal cell carcinoma
RELN: Reelin
SG: Stress granule
shRNA: Short hairpin RNA
siRNA: Small interfering RNA
SPP1: Secreted phosphoprotein 1
TNM: Tumor, node, metastasis
YBX1: Y-box binding protein 1
