# Supplementary figures and images for: The interaction of YBX1 with G3BP1 promotes renal cell carcinoma cell metastasis via YBX1/G3BP1-SPP1- NF-κB signaling axis
Source: J Exp Clin Cancer Res. 2019 Sep 3;38:386. doi: 10.1186/s13046-019-1347-0 (PMC6720408; doi:10.1186/s13046-019-1347-0)

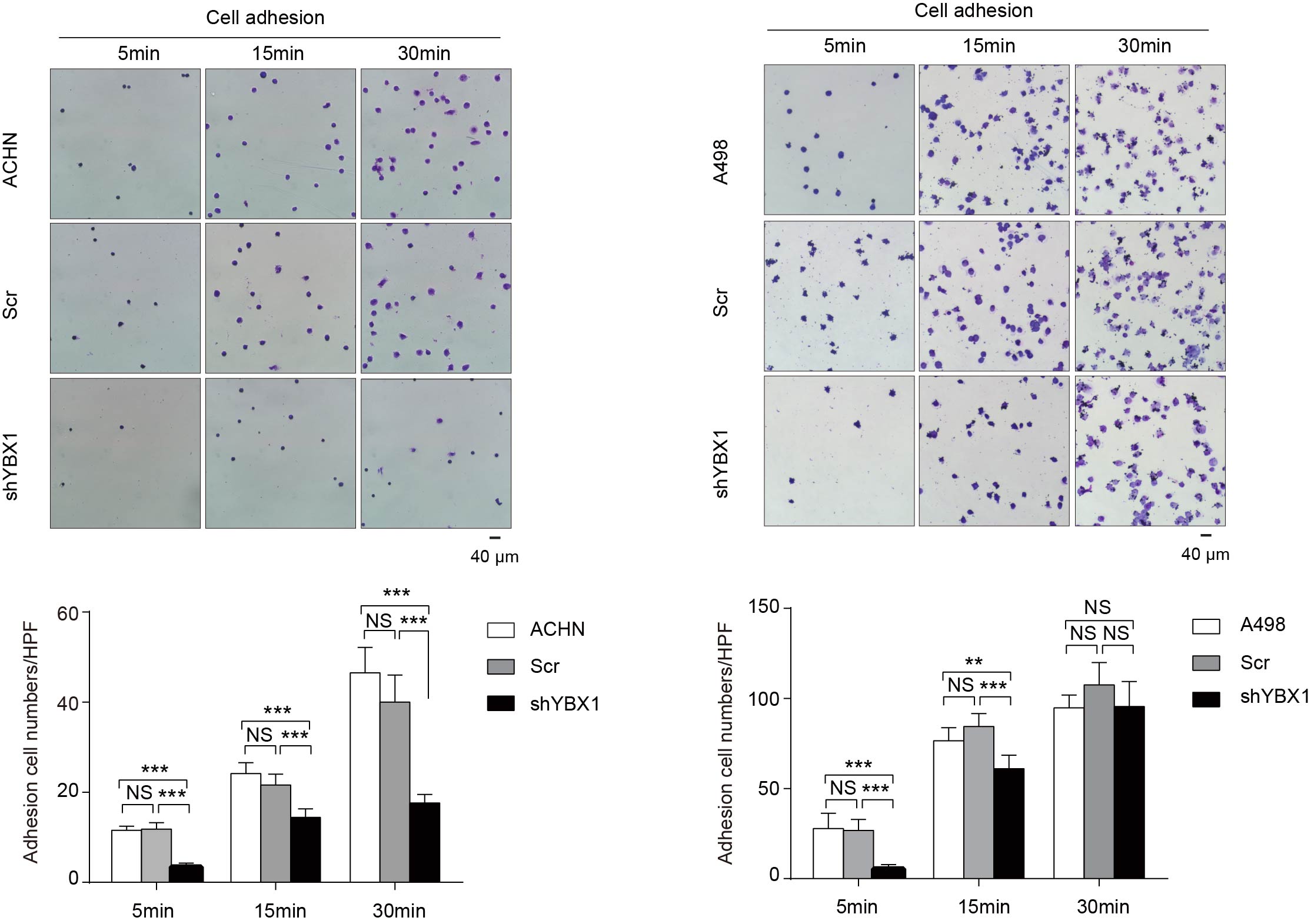

Supplement: Supplementary file 1 — Figure S1. YBX1 promotes the adhesion ability of RCC cells. (A) Depletion of YBX1 decreased ACHN cells adhesion ability at 5 min, 15 min and 30 min. (B) Depletion of YBX1 significantly decreased A498 cells adhesion ability at 5 min, 15 min. Upper panel: representative microscopic images. Lower panel: quantitative analysis. The data were presented as mean ± SD of three independent experiments, and five random microscopic fields were acquired in each experiment for quantification. Statistically significant differences were indicated: **, p < 0.01; ***, P < 0.001. NS: no significant difference. (JPG 242 kb) [file 13046_2019_1347_MOESM1_ESM.jpg]

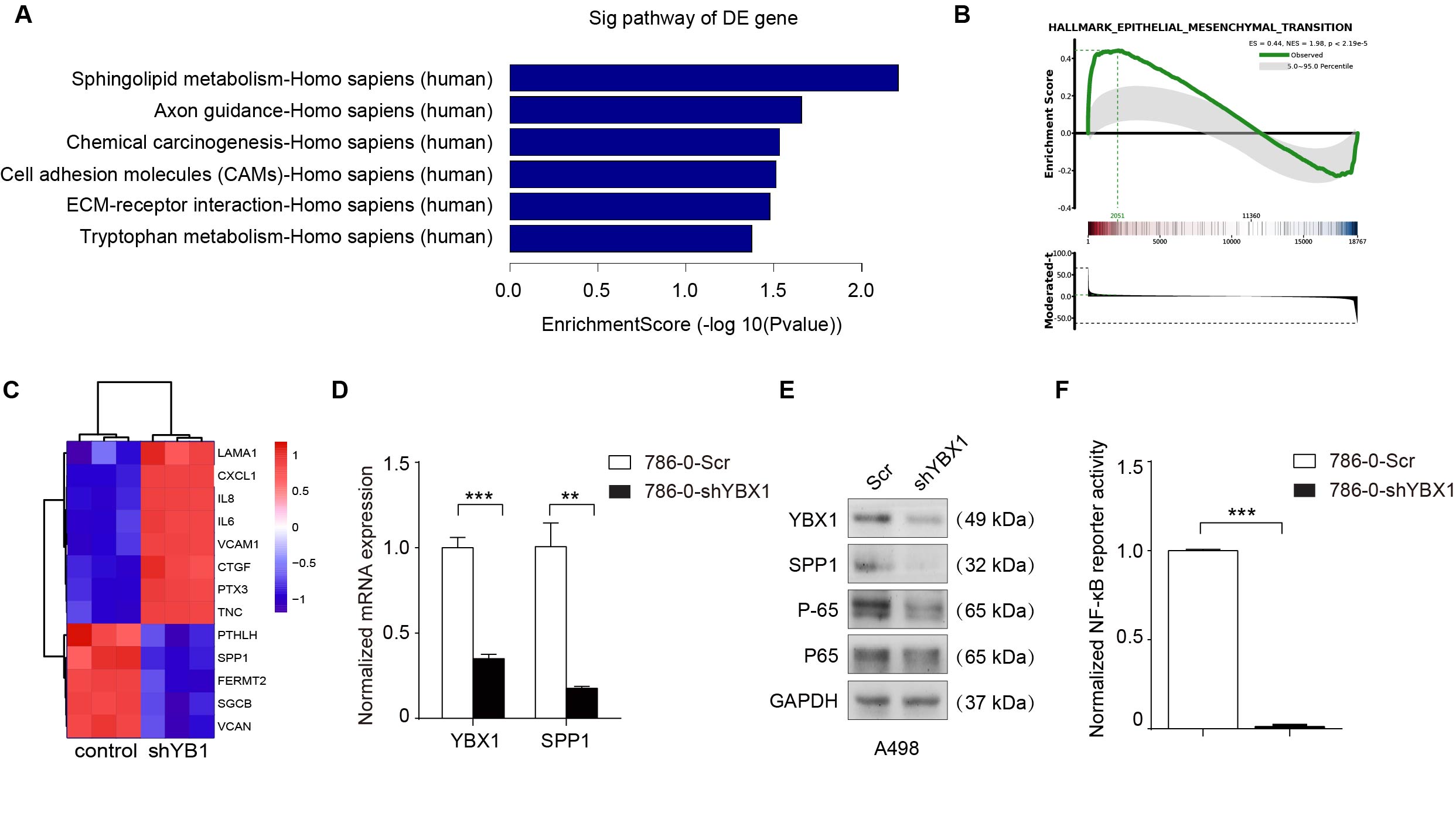

Supplement: Supplementary file 2 — Figure S2. YBX1 regulates SPP1 to activate downstream NF-κB signaling pathway in RCC cells. (A) The genes regulated by YBX1 were enriched in sphingolipid metabolism, axon guidance, chemical carcinogenesis, cell adhesion molecules (CAMs), ECM-receptor interaction, tryptophan metabolism pathways. (B) Gene set enrichment analysis was performed to identify genes that have positive or negative correlations with YBX1 expression. Enrichment plots showed significant correlation of the EMT process. (C) Heat maps for genes upregulated or downregulated by YBX1 knockdown in EMT process. (D) YBX1 knockdown decreased the level of the SPP1 mRNA in 786–0 cells. (E) The expressions of YBX1, SPP1, p-p65 (Ser536), and total p65 were examined by western blot in A498-Scr and A498-shYBX1 cells. (F) 786–0 cells stably knockdown YBX1 and control cells were transiently with NF-κB pathway firefly luciferase reporter together with internal control Renilla luciferase reporter (pRL-TK) vector. Then, luciferase activity was measured using a dual-Luciferase Reporter Assay System (Promega). Statistically significant differences were indicated: **, p < 0.01; ***, P < 0.001. NS: no significant difference. (JPG 241 kb) [file 13046_2019_1347_MOESM2_ESM.jpg]

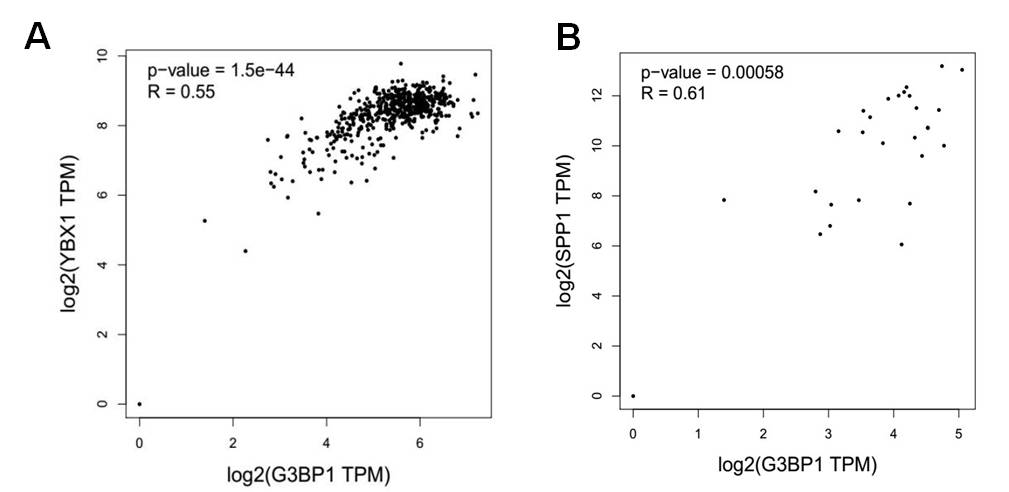

Supplement: Supplementary file 3 — Figure S3. Correlation between the expression of YBX1, G3BP1 and SPP1 from LinkedOmics database. (A) YBX1 was positively correlated with G3BP1. (B) G3BP1 was positively correlated with SPP1. (JPG 28 kb) [file 13046_2019_1347_MOESM3_ESM.jpg]

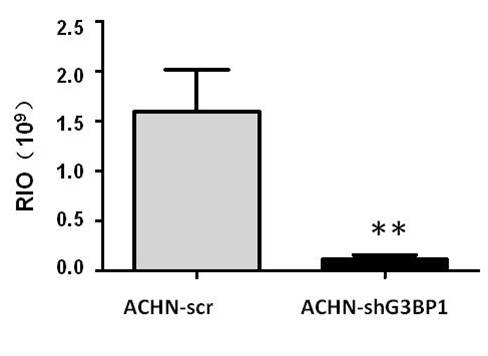

Supplement: Supplementary file 4 — Figure S4. The bioluminescence intensity of RCC in G3BP1 knockdown group and control group in vivo. (JPG 11 kb) [file 13046_2019_1347_MOESM4_ESM.jpg]
