# Supplementary material for: The interaction of YBX1 with G3BP1 promotes renal cell carcinoma cell metastasis via YBX1/G3BP1-SPP1- NF-κB signaling axis
Source: J Exp Clin Cancer Res. 2019 Sep 3;38:386. doi: 10.1186/s13046-019-1347-0 (PMC6720408; doi:10.1186/s13046-019-1347-0)
Supplement: Supplementary file 5 — Table S1. Expression of YBX1, G3BP1 and SPP1 in RCC tissues. (DOCX 13 kb) [file 13046_2019_1347_MOESM5_ESM.docx]

**Table S1** Expression of YBX1, G3BP1 and SPP1 in RCC tissues

|  |  |  | YBX1 expression | | *p* Value | G3BP1 expression | | *p* Value | SPP1 expression | | *p* Value |
| --- | --- | --- | --- | --- | --- | --- | --- | --- | --- | --- | --- |
|  |  | N | - | + |  | - | + |  | - | + |  |
| Gender | Male | 43 | 14 | 29 | 0.550 | 14 | 29 | 0.294 | 13 | 30 | 0.704 |
|  | Female | 17 | 4 | 13 |  | 8 | 9 |  | 6 | 11 |  |
| Age | <60 | 21 | 5 | 16 | 0.443 | 7 | 14 | 0.694 | 4 | 17 | 0.154 |
|  | ≥60 | 39 | 13 | 26 |  | 15 | 24 |  | 15 | 24 |  |
| Tumor size | <4 | 12 | 3 | 9 | 0.833 | 3 | 9 | 0.605 | 3 | 9 | 0.835 |
|  | >4, ≤7 | 14 | 5 | 9 |  | 5 | 9 |  | 5 | 9 |  |
|  | >7 | 34 | 10 | 24 |  | 14 | 20 |  | 11 | 23 |  |
| TNM stage | T1–2 | 42 | 17 | 25 | 0.006 | 21 | 21 | 0.001 | 17 | 25 | 0.034 |
|  | T3–4 | 18 | 1 | 17 |  | 1 | 17 |  | 2 | 16 |  |
| Fuhrman grade | I, II | 37 | 15 | 22 | 0.041 | 18 | 19 | 0.026 | 18 | 19 | 0.000 |
|  | III, IV | 23 | 3 | 20 |  | 4 | 19 |  | 1 | 22 |  |
